# Personality, self-esteem, familiarity, and mental health stigmatization: a cross-sectional vignette-based study

**DOI:** 10.1038/s41598-022-14017-z

**Published:** 2022-06-20

**Authors:** Sahar Steiger, Julia F. Sowislo, Julian Moeller, Roselind Lieb, Undine E. Lang, Christian G. Huber

**Affiliations:** 1 University Psychiatric Clinics Basel, University of Basel, Wilhelm Klein-Str. 27, 4012 Basel, Switzerland; 2 Division of Clinical Psychology and Epidemiology, Department of Psychology, University of Basel, Missionsstr. 60/62, 4055 Basel, Switzerland

**Keywords:** Psychology, Health care

## Abstract

There has been little research exploring the relationship between personality traits, self-esteem, and stigmatizing attitudes toward those with mental disorders. Furthermore, the mechanisms through which the beholder’s personality influence mental illness stigma have not been tested. The aim of this study is to examine the relationship between Big Five personality traits, self-esteem, familiarity, being a healthcare professional, and stigmatization. Moreover, this study aims to explore the mediating effect of perceived dangerousness on the relationship between personality traits and desire for social distance. We conducted a vignette-based representative population survey (*N* = 2207) in the canton of Basel-Stadt, Switzerland. Multiple regression analyses were employed to examine the associations between personality traits, self-esteem, familiarity, and being a healthcare professional with the desire for social distance and perceived dangerousness. The mediation analyses were performed using the PROCESS macro by Hayes. Analyses showed associations between personality traits and stigmatization towards mental illness. Those who scored higher on openness to experience (*β* = − 0.13, *p* < 0.001), (*β* = − 0.14, *p* < 0.001), and those who scored higher on agreeableness (*β* = − 0.15, *p* < 0.001), (*β* = − 0.12, *p* < 0.001) showed a lower desire for social distance and lower perceived dangerousness, respectively. Neuroticism (*β* = − 0.06, *p* = 0.012) was inversely associated with perceived dangerousness. Additionally, high self-esteem was associated with increased stigmatization. Personal contact or familiarity with people having mental disorders was associated with decreased stigmatization. Contrarily, healthcare professionals showed higher perceived dangerousness (*β* = 0.04, *p* = 0.040). Finally, perceived dangerousness partially mediated the association between openness to experience (indirect effect = −  .57, 95% CI [− .71, − 0.43]) as well as agreeableness (indirect effect = − 0.57, 95% CI [− 0.74, − 0.39]) and desire for social distance. Although the explained variance in all analyses is < 10%, the current findings highlight the role of personality traits and self-esteem in areas of stigma. Therefore, future stigma research and anti-stigma campaigns should take individual differences into consideration. Moreover, the current study suggests that perceived dangerousness mediates the relationship between personality traits and desire for social distance. Further studies are needed to explore the underlying mechanisms of such relationship. Finally, our results once more underline the necessity of increasing familiarity with mentally ill people and of improving the attitude of healthcare professionals towards persons with mental disorders.

## Introduction

Despite several attempts to promote destigmatization, mental illness stigma has been relatively stable across the last decades. Exclusion, rejection, and discrimination of people with mental illness is widespread^1^, and has multiple negative consequences for individuals with mental illness^2^, including reduced treatment-seeking behavior, decreases in self-esteem^3^, and poor adherence to medication^2^. Perception of dangerousness^4,5^ and desire for social distance^6^ are most common areas of research for identifying the correlates of stigmatization that affect the outcomes of persons with mental illness. Evidence from various studies has shown that the perception of dangerousness increases the tendency to socially distance oneself from people with mental disorders^7^. The perception of dangerousness has also been found to mediate the relationship between disorder type and social distance^8^. Moreover, the impact of familiarity with mental disorders towards individuals with mental illness is well established in stigma research. Being familiar with people having a mental disorder has been found to reduce the desire for social distance^9^.

Although many studies have examined social distance, perceived dangerousness, and familiarity as correlates of stigmatization, only limited research has explored personality traits and individual differences that might be associated with stigmatization of mental illness. Canu et al. explored the social appraisal of adults with attention deficit hyperactivity disorder (ADHD) among college students and revealed that agreeableness, extraversion, and conscientiousness were significantly associated with a desire to engage with people with ADHD^10^. Using the Big Five model of personality^11^, Brown showed that openness and agreeableness were negatively associated with stigmatization towards mental illness^12^. In addition, research on the role of self-esteem in the stigmatization process is scarce. For instance, individuals with high self-esteem interact in more antagonistic ways^13^. Yet, it is unclear whether self-esteem is associated with stigmatization towards mental illness.

To date, no studies have been published that examine the potential mediating effect of perceived dangerousness on the relationship between personality traits and stigmatizing attitudes toward those with mental disorders using data from a representative population survey. Thus, the current study aims to (1) examine the relationship between Big Five personality traits, self-esteem, and familiarity with stigmatization towards mental illness; and (2) to explore whether perceived dangerousness mediates the relationship between personality traits and stigmatization. The current study is exploratory in nature and aims to replicate and advance previous findings that have examined the relationship between personality traits and stigmatization towards mental disorders.

## Methods

### Sample and procedure

Data for the current study stem from a vignette-based representative population survey on psychiatric service use and stigmatization that was conducted from autumn 2013 to spring 2014 among citizens of Basel, Switzerland. A sample of 10,000 individuals was randomly drawn from the cantonal resident register and was mailed study material. To be eligible, participants had to have been registered in a private household in the municipality of Basel, Bettingen, or Riehen for a minimum of 2 years, had to be aged between 18 and 65 years, and had to have sufficient knowledge of the German language. This study was approved by the local ethics committee (Ethikkommission Nordwest-und Zentralschweiz, EKNZ 2014-394) and conducted according to the Declaration of Helsinki. Informed consent was obtained from all the study participants and they agreed to return the completed survey material. Participants were informed about the scope of the study and their rights in an accompanying letter. An email address and hotline telephone number were provided in case the participants needed additional information.

The final sample consisted of 2207 individuals (61.5% female, 44.7% single, 65% Swiss citizens, 16% dual citizenship, 19.0% other nationalities), reflecting a response rate of 22.1%. The mean age of the participants was 43.4 years (*SD* = 13.4). A total of 6.2% had completed only the 9 years of schooling obligatory in Switzerland, 51.3% had completed secondary education (approximately 12 years), and 42.0% had a university degree.

To assess the representativeness of our sample, respondent characteristics were compared to official census data as published in the statistical Almanac of Basel-City^14^. However, this comparison has to be interpreted with caution, as the data available from the statistical almanac represent the whole population of Basel-City without the restrictions posed by our in- and exclusion criteria. At the end of 2013, 191,606 persons were registered in Basel-City. Fifty-two percent were of female gender, 67.0% were Swiss citizens, and 45.7% were single. Mean age was 42.9 years. Eighteen percent had completed obligatory school, 48.6% secondary education, and 32.5% higher/university education. The comparison shows that questionnaires were sent out to over 5.2% of the population. The study sample represents more than 1.2% of the total population and can be assumed to be representative regarding age, nationality, marital status, and living situation. However, there seems to be an overrepresentation of women and of persons with higher education in our sample.

### Study material

Study material consisted of written vignettes and questionnaires. The vignettes were published as supplemental material to Sowislo et al.^6^. Apart from sociodemographic variables, the questionnaires measured desire for social distance and perceived dangerousness as indicators for stigmatization, familiarity with mental illness, approval of coercion, and personality traits. Vignettes presented a fictitious character and depicted either a psychiatric disorder of the character (case vignette) or a clinic where the character had been admitted to (clinic vignette). Within the vignettes, the gender and endangering behavior of the fictitious patient were systematically varied. Between the case vignettes, the type of psychiatric disorder was systematically varied, which either described a case of acute psychotic disorder, a case of alcohol dependency, or a case of borderline personality disorder. None of these were labelled directly, but they had symptoms fulfilling the DSM-V criteria^15^ for the respective disorder. Case vignettes were constructed based on vignettes used in previous stigma research^16^. Apart from these characteristics, all other information was kept constant between the vignettes to eliminate potential confounders.

Moreover, between the clinic vignettes, the type of psychiatric service institution to which the fictitious character was admitted was also systematically varied. Vignettes either described a general hospital that included a psychiatric unit, or a psychiatric hospital, or a psychiatric hospital that included a forensic unit.

The current study is the fourth in a series that examines stigmatization related to type of psychiatric symptoms, psychiatric service use and approval of coercion in the public perceptions. The previous studies had examined the desire for social distance^6^, the perceived dangerousness^4^ and the prediction of the approval of coercive measures^17^. Further studies on case-related and general approval of coercive measures^18^ and the attitude of the general public in Basel concerning the differential use of coercive measures^19^ are currently in the review process.

### Measures

Desire for social distance was measured using a modification^20^ of the Bogardus Social Distance Scale^21^. We used the German translation of the scale of social distance^22^, which has been used in several studies, and for which unidimensionality, construct validity, predictive validity, and sensitivity to change have been repeatedly shown^23^. The scale consists of seven items asking to what degree the respondent would accept each of the following social relationships with the stigmatized person: sublessee, co-worker, neighbor, caretaker of one’s child, spouse of a family member, and member of the same social circle. Responses were made on a 4-point scale, with lower values indicating greater acceptance of the person in the vignette (i.e., a lower desire for social distance). In our study, the reliability (Cronbach's alpha) of the seven items was 0.86.

Perceived dangerousness was measured with the dangerousness scale^24,25^. The scale consists of eight items that assess individual beliefs about the dangerousness of the fictitious person in the vignette. Responses were made on a 4-point scale and a composite (with higher values indicating higher perceived dangerousness) was derived by totalling the sum of all items. The reliability (Cronbach's alpha) of the scale in this study was 0.79.

Personality was assessed using the Big Five Inventory 10 (BFI-10)^26^, in which the Big Five Inventory 44 (BFI-44)^27^ was abbreviated to a 10-item version, with 2 items measuring each of the Big Five personality traits (i.e., extraversion, agreeableness, conscientiousness, neuroticism, and openness to experience). The mean coefficient alpha of the BFI-44 is high (α = 0.83), as is the 3-month test–retest reliability (*r* = 0.85). The BFI-10 scales captured 70% of the full BFI variance and retained 85% of the retest reliability^26^. Participants were required to read items such as ‘I see myself as someone who is generally trusting’, ‘I see myself as someone who gets nervous easily’, and then rate how accurately each item describes themselves using a 5-point Likert scale, with ‘1 = very accurate’ and ‘5 = very inaccurate’. An average score was calculated for each personality trait, with a higher score representing a higher endorsement of the personality trait.

Self-esteem was measured with the Single-Item Self-Esteem Scale: “I have high self-esteem”^28^, which applies a 5 -point Likert scale (agree strongly to disagree strongly).

Familiarity with mental illness was examined with three items, similar to the approach of Angermeyer et al.^7^, asking whether psychiatric treatment had been undergone by (1) the participant, (2) a family member of the participant, or (3) a friend of the participant. If the criteria for multiple categories were fulfilled, we chose the one indicating the highest familiarity. In addition, participants were asked if they were healthcare professionals.

### Statistical analysis

All statistical analyses were conducted using the SPSS 24 statistical package for Windows (IBM Corporation, Armonk, NY, USA). Descriptive analysis was performed for socio-demographics and other variables. Mean and standard deviation (SD) were calculated for continuous variables, while for categorical variables their frequencies and percentage were presented. First, we examined the bivariate associations between socio-demographics including age, gender, and education level with social distance and perceived dangerousness using a linear regression analysis. Second, multiple regression analyses with desire for social distance and perceived dangerousness as dependent variables were conducted. BFI personality traits (extraversion, neuroticism, openness, conscientiousness, agreeableness), self-esteem, familiarity, respondent being a healthcare professional, and the significant socio-demographics from the first analysis were entered as independent variables. As a third step, we conducted a mediation analysis with perceived dangerousness as a mediator, desire for social distance as a dependent variable, and the significant BFI traits from the first analysis as independent variables. The mediation analyses were performed using the PROCESS macro by Hayes^29^, which uses ordinary least squares regression, yielding unstandardized path coefficients for total, direct, and indirect effects. Effects were deemed significant when the confidence interval did not include zero. For all other analyses, the level of significance was set at *p* ≤ 0.05.

### Ethics committee approval

This study was approved by the local ethics committee (EKNZ 2014-394) and conducted according to the Declaration of Helsinki.

## Results

### Desire for social distance

The linear regression analyses showed bivariate associations between gender (*β* = − 0.08, *p* < 0.001) and education level (*β* = − 0.06 *p* = 0.005) with social distance. The regression model containing all predictors was significant (*N* = 2056, *F* (12, 2044) = 15.38, *p* < 0.001). It explained 7.7% (adjusted *R* square) of the variance in the desire for social distance.

The multiple regression analysis revealed that openness to experience (*β* = − 0.13, *p* < 0.001), and agreeableness (*β* = − 0.15, *p* < 0.001) were negatively associated with desire for social distance, which means that an increase in the level of openness and agreeableness was linked with reduced desire for social distance (see Table 1). There were no significant associations between other Big Five variables and desire for social distance. Self-esteem was positively associated with desire for social distance (*β* = 0.09, *p* < 0.001), which indicates that people with higher self-esteem might express a stronger desire for social distance from people with mental illnesses.

**Table 1:** Multiple regression analyses for social distance.

	Social distance			
	*B*	*SE*	*β*	*p*
**Personality trait**				
Extraversion	−0.125	0.107	− 0.027	0.243
Neuroticism	0.145	0.120	0.030	0.227
Openness	−0.636	0.112	− 0.126	< 0.001
Conscientiousness	0.225	0.131	0.038	0.086
Agreeableness	−0.879	0.128	− 0.148	< 0.001
Self esteem	0.436	0.117	0.093	< 0.001
**Familiarity**				
Self	− 2.055	0.367	− 0.215	< 0.001
Family	− 1.588	0.363	− 0.164	< 0.001
Friends	− 1.280	0.376	− 0.120	< 0.001
Healthcare professional	0.326	0.222	0.032	0.142
**Gender**				
male vs. female	− 0.419	0.210	− 0.045	0.046
Education level	− 0.111	0.054	− 0.044	0.042
Constant	15.578	1.086		< 0.001

*B,* unstandardized regression weight; *β,* standardized regression weight; *SE*, standard error; *p*, *p*-Value.

Concerning familiarity, a participant her-/himself ((*β* = − 0.22, *p* < 0.001), a family member (*β* = − 0.16, *p* < 0.001), or a friend (*β* = − 0.12, *p* < 0.001) having undergone psychiatric treatment were significantly associated with a lower desire for social distance, indicating that individuals who have experience with mental disorders stigmatize people with mental illness to a lesser degree. Being a healthcare professional was not significantly associated with desire for social distance. Gender (*β* = − 0.05, *p* = 0.046), and education level (*β* = − 0.04, *p* = 0.042) were negatively associated with social distance. Regarding gender, female participants in our study reported significantly less stigmatization than did the men.

### Perceived dangerousness

There linear regression analyses did not reveal significant bivariate associations between age, gender, and education level with perceived dangerousness.

The total model containing all predictors was significant (*N* = 2065, *F* (10, 2055) = 19.87, *p* < 0.001). It explained 8.4% (adjusted *R* square) of the variance in perceived dangerousness.

The multiple regression analysis revealed negative associations between openness to experience (*β* = − 0.14, *p* < 0.001), agreeableness (*β* = − 0.12, *p* < 0.001), neuroticism (*β* = − 0.06, *p* = 0.012), and perceived dangerousness, which indicates that people with higher scores in openness to experience, agreeableness, and neuroticism reported lower perceived dangerousness. Neither conscientiousness nor extraversion were significantly linked to perceived dangerousness. On the contrary, there was a positive relationship between self-esteem (β = 0.07, p = 0.003) and perceived dangerousness (see Table 2).

**Table 2:** Multiple regression analyses for perceived dangerousness.

	Perceived dangerousness			
	*B*	*SE*	*β*	*p*
**Personality trait**				
Extraversion	− 0.043	0.105	− 0.009	0.683
Neuroticism	− 0.309	0.123	− 0.061	0.012
Openness	− 0.742	0.114	− 0.142	< 0.001
Conscientiousness	0.097	0.0132	0.016	0.464
Agreeableness	− 0.761	0.132	− 0.124	< 0.001
Self esteem	0.355	0.119	0.074	0.003
**Familiarity**				
Self	− 2.854	0.374	− 0.287	< 0.001
Family	− 2.217	0.369	− 0.221	< 0.001
Friends	− 1.597	0.384	− 0.144	< 0.001
Healthcare professional	0.462	0.225	0.044	0.040
Constant	14.072	0.789		< 0.001

*B,* unstandardized regression weight; *β,* standardized regression weight; *SE*, standard error; *p*, *p*-value.

Concerning familiarity, a participant her-/himself (*β* = -0.29, *p* < 0.001), a family member (*β* = − 0.22, *p* < 0.001), or a friend (*β* = − 0.14, *p* < 0.001) having undergone psychiatric treatment were inversely associated with perceived dangerousness, indicating that the more familiar the respondents are with mental illness, the less dangerous they believe the person depicted in the vignette to be. On the other hand, healthcare professionals were more likely to perceive the person depicted in the vignette as dangerous (*β* = 0.04, *p* = 0.040).

### Mediation analyses

**Figure 1:**
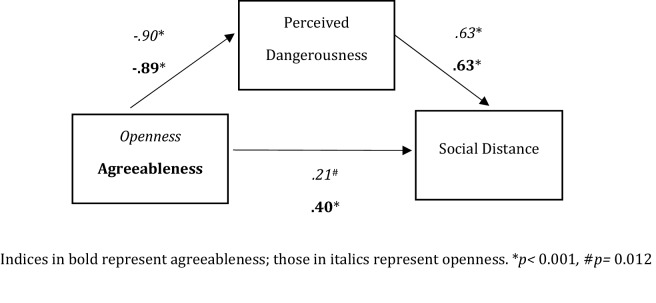
Mediating effect of perceived dangerousness on the relationship between personality and desire for social distance. Indices in bold represent agreeableness; those in italics represent openness. *p < 0.001*,* ^#^*p* = 0.012.

Mediation analyses were performed to analyze whether the association between personality and desire for social distance were mediated by perceived dangerousness (see Fig. 1). An effect of openness (*B* = − 0.78, *p* < 0.001), and agreeableness (*B* = − 0.96, *p* < 0.001) on desire for social distance was found. After entering the mediator into the model, openness predicted perceived dangerousness significantly (*B* = − 0.90, *p* < 0.001), which in turn predicted the desire for social distance (*B* = 0.63, *p* < 0.001). Respectively, agreeableness predicted perceived dangerousness significantly (*B* = 0.90, *p* < 0.001), which in turn predicted desire for social distance (*B* = 0.63, *p* < 0.001).

Additionally, we found that the relationship between openness to experience and desire for social distance (*B* = − 0.21, *p* = 0.012) remained significant after we have included perceived dangerousness in the model. This was also true for the relationship between agreeableness and desire for social distance (*B* = − 0.40, *p* < 0.001). Thus, the mediation analyses indicated that perceived dangerousness partially mediated the relationship between openness (indirect effect = − 0.57, 95% CI [− 0.71, − 0.43]) as well as agreeableness (indirect effect = − 0.57, 95% CI [− 0.74, − 0.39]) and desire for social distance (see Table 3).

**Table 3:** Total effect, direct effect, and indirect effect of personality on the desire for social distance.

	Total effect	*p*	Direct effect	*p*	Indirect effect	*CI*
Openness	− 0.777	< 0.001	− 0.207	0.012	− 0.569	− 0.713, − 0.429
Agreeableness	− 0.962	< 0.001	− 0.395	< 0.001	− 0.567	−0.735, −0.394

Indirect effect was deemed significant when the confidence interval did not include zero.

We have also explored the potential mediation effects of perceived dangerousness on the relation between social distance, the other three personality traits and self-esteem, none of them were significant.

## Discussion

This study examined the role of personality, self-esteem, familiarity with mental illness, and being a healthcare professional on stigmatization towards individuals with mental illness. Moreover, this study investigated whether the effect of personality on the desire for social distance as an indicator of stigmatization may operate indirectly via the influence of perceived dangerousness.

Our findings indicated that agreeableness and openness to experience are negatively associated with mental illness stigmatization. These findings are in line with Yuan et al.^30^. They also found that agreeableness and openness to experience were negatively associated with stigmatization towards mental illness using a vignette-based approach. People scoring higher on agreeableness are generally well-natured, cooperative, and concerned for others^11^. Additionally, agreeable people are empathetic, altruistic, and show great kindness and gentleness^31^. This may indicate that they treat people with mental disorders with consideration, compassion, trust, and are less likely to perceive them as dangerous or to exhibit a desire for social distance. Openness to experience implies creativity, curiosity, as well as self-determination^32^. Individuals who score highly on openness to experience tend to be open-minded, unconventional, and imaginative^11^. These features can help them to express greater social comfort and more understanding in interactions with mentally ill individuals.

In addition, other research found that increased levels of empathy and perspective-taking lead to less prejudice^33^. If we assume the similarities between prejudice and stigma, we thus surmise that people scoring higher on agreeableness (helpful, altruistic, sympathetic) and openness (open-minded, receptive, curious) may exhibit higher empathy and greater perspective taking towards mentally ill people and could therefore be less likely to have negative attitudes about them or to endorse the stereotype of dangerousness. However, it is unclear whether the relationship between personality and prejudice might be extended and generalized to mental illness stigmatization. Future studies should investigate this topic.

In our analyses, neuroticism was negatively associated with perceived dangerousness. Individuals with a high degree of neuroticism tend to be anxious, moody, and insecure^11^. A meta-analysis demonstrated that higher levels of neuroticism are related to a broad range of clinical mental disorders such as depression and anxiety^34^. The background of the association between neuroticism and decreased perceived dangerousness is currently unclear. We speculate that persons with high neuroticism might increasingly attribute themselves as being affected by mental health conditions, or that they might be reluctant to attribute dangerousness to persons with mental illness as this opinion is less socially desirable.

Our findings also emphasized the significant role of familiarity in reducing mental illness stigmatization, which is well established in stigma research^7,9^. All categories of familiarity were associated with less desire for social distance and less perceived dangerousness. However, the context of familiarity and the selection of encountered persons seem to play an important role: other than having contact with persons with mental illness in a private context, being a health care professional was associated with exhibiting more stigma towards mentally ill people. In line with this finding, a previous study in a large sample of Swiss mental health professionals found that health care professionals, compared with the general population, hold negative stereotypes and stigmatizing attitudes towards people with mental illness^35^. These stigmatizing attitudes might have a negative impact on patients. Findings from a systematic review suggested that contact mental-health-care professionals have with people with mental illness does not reduce stigma as does social contact such as with friends or family members^36^. A meta-analysis by Corrigan et al. showed that direct contact was the most effective anti-stigma intervention for adults, whereas educational approaches were more beneficial for adolescents^37^. Selecting of healthcare professionals with higher levels of openness and agreeableness or developing interventions that would increase their level of Openness and Agreeableness might enhance their ability to work with patients with mental illness. Brown^12^ suggests that using personality traits as a screening tool for health care providers may result in less stigma. Integrating personality modification interventions into the educational programs for health care professionals might reduce stigma. Future studies are still needed to explore interventions based on personality traits.

The association between high self-esteem and increased mental illness stigmatization warrants further research. Some studies differentiate between implicit and explicit self-esteem^38^. For instance, individuals with high explicit self-esteem but low implicit self-esteem are more likely to discriminate ethnically. Stigmatization can be used as an effective technique to protect self-esteem^39^. There is also an association between self-esteem and narcissism^40^, and persons with a high level of narcissism tend to be more prejudiced^41^.

Finally, personality may influence the desire for social distance not only directly but also indirectly by reducing perceived dangerousness. Perceived dangerousness conveys only part of the effect of personality on the desire for social distance. This finding should be further explored in future studies to improve insight in the mechanisms underlying the effect of personality on the stigmatization process. According to Corrigan^42^, the perception of dangerousness of persons with mental disorders leads to fear and then to social avoidance. On the other hand, high openness and agreeableness might lead to perceiving persons with mental disorders as less dangerous. This, in turn, may tend to decrease anxiety and subsequently reduce desire for social distance.

### Limitations

This study has some limitations. Firstly, the low response rate of 22.1% might account for selection and nonresponse biases (e.g., reflecting increased participation of women and of persons with higher education). Secondly, the measurement of desire for social distance and perceived dangerousness is based on hypothetical scenarios, and therefore might be different from the respondents’ real-life behavior. Moreover, familiarity was measured with three single items, which might threaten the internal validity of the domain meant to be measured. Furthermore, personality traits were measured in this study with short scales (two items). This could indicate that the personality dimensions might not be accurately represented. Thus, this research should be replicated in future studies that use full-length Big Five measures. Thirdly, this study was exploratory in nature and based on correlational analyses. Thus, no causal interpretation of the findings can be drawn. Finally, the regression models only explained a limited amount of variance regarding desire for social distance and perceived dangerousness—other variables were not available for the current analyses and therefore might influence these outcomes to a considerable degree and should be included in further studies on the topic.

## Conclusion

The current analyses support the hypothesis of a relationship between personality, self-esteem, and stigmatization towards mental illness. People high in openness to experience and agreeableness score lower on desire for social distance and perceived dangerousness. Anti-stigma interventions should be aware that public perceptions of the dangerousness of mentally ill people are influenced by personality traits, and that perceived dangerousness increases stigmatization, acting as a partial mediator of the effects of openness to experience and agreeableness on the desire for social distance. In addition, the results underline the necessity of increasing familiarity with persons with mental illness in the general population, and of improving the attitude of healthcare professionals towards persons with mental disorders.

## Data Availability

The data that support the findings of this study are available from the corresponding author upon request.
